# Preconditioned hyperbaric oxygenation protects skin flap grafts in rats against ischemia/reperfusion injury

**DOI:** 10.3892/mmr.2014.2064

**Published:** 2014-03-24

**Authors:** NAN KANG, YONG HAI, FANG LIANG, CHUN-JIN GAO, XUE-HUA LIU

**Affiliations:** 1 Department of Orthopaedics, Beijing Chaoyang Hospital, Capital Medical University, Beijing 100020, P.R. China; 2 Department of Hyperbaric Oxygen, Beijing Chaoyang Hospital, Capital Medical University, Beijing 100020, P.R. China

**Keywords:** hyperbaric oxygen preconditioning, skin flap, high mobility group protein 1, nuclear factor-κB

## Abstract

Hyperbaric oxygen (HBO) therapy is an effective therapy for ischemia/reperfusion (I/R) injury of the brain, small intestine, testes and liver. However, the detailed molecular mechanisms underlying the effect of HBO therapy remain undetermined. In the current study, the hypothesis that preconditioning rats with HBO protects grafted skin flaps against subsequent I/R injury was investigated. In addition, the molecular mechanisms underlying HBO therapy were characterized by analyzing the roles of the following important inflammatory factors: High mobility group protein 1 (HMGB1) and nuclear factor-κ B (NF-κB). A total of 40 rats were randomly divided into the following five groups: (i) Sham surgery (SH); (ii) ischemia followed by reperfusion 3 days following surgery (I/R3d); (iii) ischemia followed by reperfusion 5 days following surgery (I/R5d); (iv) HBO preconditioning (HBO-PC) and ischemia followed by reperfusion 3 days following surgery (HBO-PC+3d); and (v) HBO-PC and ischemia followed by reperfusion 5 days following surgery (HBO-PC+5d). For the surgical procedure, all pedicled skin flaps were first measured and elevated (9×6 cm). The feeding vessels of the skin flaps were subsequently clamped for 3 h and released to restore blood flow. The rats in the HBO-PC+3d and HBO-PC+5d groups received 1 h HBO for 3 and 5 consecutive days, respectively, prior to surgery. Following surgery, the rats were euthanized, and grafted tissues were collected for western blotting and immunohistochemistry. HBO-PC increased blood perfusion in epigastric skin flaps and attenuated I/R injury following skin flap graft. Additionally, the elevated expression of HMGB1 and NF-κB proteins during I/R injury was attenuated by HBO-PC treatment. HBO-PC may therefore be applied to reduce I/R injury and improve the survival rate of grafted skin flaps. The molecular mechanisms underlying the effect of HBO therapy are associated with the attenuation of inflammatory responses.

## Introduction

Skin flap grafting is a form of transplantation widely used in plastic surgery. However, ischemia/reperfusion (I/R) injury is the main factor which reduces the survival rate of flaps following grafting. In addition, skin necrosis may occur following surgery (1–4). Studies (5,6) have previously reported that hyperbaric oxygen preconditioning (HBO-PC) may induce ischemia tolerance, which provides protection against subsequent I/R injury. One of the first studies to examine the effects of HBO-PC on I/R injury was conducted on the gerbil hippocampus (7) followed by the small intestine, testes and liver. Most recently, Wang *et al* (8) designed a series of experiments to confirm that HBO-PC reduces cerebral I/R injury by stimulating autophagy in neurocytes. These studies suggest that HBO-PC may potently protect against subsequent I/R injury in a variety of tissues and organs. However, whether HBO-PC protects grafted skin flaps against I/R injury, as well as the underlying mechanisms, remains unknown.

High mobility group box 1 (HMGB1) and nuclear factor-κ B (NF-κB) are two important inflammatory response mediators involved in I/R pathogenesis. HMGB1 mediates cytokine release and inflammation, and causes tissue damage in I/R injury by activating innate immunity via the NF-κB-activated signal transduction pathway (9–11). NF-κB is a transcriptional factor that regulates the expression of pro-inflammatory cytokines, including interleukin (IL)-1, IL-6, inducible nitric oxide synthase (iNOS) and tumor necrosis factor (TNF). NF-κB is well known to exhibit a critical role in cell survival (12).

In the current study, the hypothesis that preconditioning rats with HBO-PC protects grafted skin flaps against subsequent I/R injury was investigated. In addition, the precise roles of two important inflammatory factors, HMGB1 and NF-κB, in this protective process were characterized.

## Materials and methods

### Experimental animals

All experiments were performed in accordance with the established ethical guidelines of the Committee for the Control and Supervision of Experiments on Animals at Capital Medical University (Beijing, China). Healthy adult male Sprague-Dawley rats (250–300 g; Capital Medical University) were maintained at 25±1.0°C in a 12/12 h light/dark cycle and allowed food and water *ad libitum*.

### Experimental groups

A total of 40 rats were randomly assigned to the following five groups: Sham surgery [SH; 21% O_2_ at 1.0 atmosphere absolute (ATA)] (n=8); ischemia followed by reperfusion 3 days following surgery (I/R3d; 21% O_2_ at 1.0 ATA; n=8); ischemia followed by reperfusion 5 days following surgery (I/R5d; 21% O_2_ at 1.0 ATA; n=8); hyperbaric oxygen preconditioning and ischemia followed by reperfusion 3 days following surgery (HBO-PC+3d; 100% O_2_ at 2.0 ATA; n=8); and hyperbaric oxygen preconditioning and ischemia followed by reperfusion 5 days following surgery (HBO-PC+5d; 100% O_2_ at 2.0 ATA; n=8).

### Epigastric pedicle skin flap model

All procedures were performed aseptically under anesthesia using intraperitoneal injections of 10% chloral hydrate (350 mg/kg). Following shaving and washing of the abdomen, the rats were fixed on wooden shelves. Single inferior epigastric vessel pedicled skin flaps were designed and elevated (9×6 cm). The right inferior epigastric artery and vein pedicle were skeletonized, while the contralateral inferior epigastric vessel was ligated by suturing, and the feeding vessels of the skin flaps were clamped using a microvascular clamp to achieve ischemia. For reperfusion, the microvascular clamp was removed 3 h later, restoring blood flow. The flaps were repositioned above a silicone sheet of the same dimensions as the flap with continuous 5–0 monofilament nylon sutures to block vascular supply other than that from the pedicle. The SH group underwent the same surgery but was not subjected to ischemia. All rats received a single intramuscular injection of penicillin sodium (0.8 mg/g) postoperatively.

### Hyperbaric oxygen preconditioning

The rats in the HBO-PC+3d and HBO-PC+5d groups were placed into a custom-made transparent acrylic plastic pressure chamber (701 Space Research Institute, Beijing, China) immediately following surgery and received 1 h HBO therapy at 2.0 ATA with 100% O_2_ twice per day (at 8 h intervals) for 3 consecutive days. Compressed air was supplied at 1 kg/cm^2^/min to 2.0 ATA/100% oxygen and maintained for 1 h. The chamber was flushed with 100% oxygen at 5 l/min to avoid carbon dioxide accumulation. Decompression was performed at 0.2 kg/cm^2^/min. During HBO exposure, the oxygen and carbon dioxide contents were monitored continuously and maintained at ≥98 and ≤0.03%, respectively. The chamber temperature was maintained between 22 and 25°C. To minimize the effects of diurnal variation, all HBO exposures began at ~8:00 AM and 4:00 PM. The rats in the SH, I/R3d and I/R5d groups were treated postoperatively with normobaric air at 1.0 ATA in 21% oxygen at an ambient temperature of 22–25°C.

### Laser Doppler perfusion imaging (LDPI)

The blood flow in the pedicled skin flaps on the epigastric zone was mapped with a high-resolution PIM-2 LDPI (Perimed Inc., North Royalton, OH, USA) with a scanning area of 70×80 mm, a high resolution, and a distance of 42.3 cm between the scanner head and the wound. The images and perfusion values were analyzed using the LDISOFT software package, version 1.5 (Perimed AB, Stockholm, Sweden).

### Histological analysis

Each flap was evaluated 3 and 5 days postoperatively. For each flap, 3–4 μm tissue blocks sectioned from the viable region were fixed in a standard manner in 10% formalin and embedded in paraffin for hematoxylin-eosin staining. Images were subsequently captured using an Olympus BX51 microscope (Olympus Optical Co., Tokyo, Japan) with a ×40 objective. According to the Zdichavsky *et al* (13) score for skin injury and Rongione *et al* (14) histological score for acute pancreatitis, the degree of microscopic injury was scored on the basis of the following histological changes: Congestion, epidermal edema and leukocyte infiltration. The severity of injury was graded for each variable as follows: No injury, 0; 1, injury ≤25% of the field; 2, injury >25% to ≤55% of the field; 3, injury >55% to ≤75% of the field; 4, diffuse injury. All evaluations were performed in a double-blinded manner.

### Immunohistochemical staining

Histological sections of tissue (3–4 μm thick) were obtained, fixed in 10% formalin, and embedded in paraffin. The sections were deparaffinized in xylene and rehydrated in ethanol, and endogenous peroxidase was blocked by immersion in methanol containing 0.3% hydrogen peroxidase for 20 min. Prior to incubation, the sections were permeabilized and blocked with normal goat serum. The sections were incubated overnight at 4°C with primary rat monoclonal antibodies (Histostain-Plus kit; Sunbio, Beijing, China). On the following day, the sections were incubated with secondary antibodies and horseradish peroxidase enzyme markers for 10–15 min followed by staining with diaminobenzidine. The slides were examined using a Nikon i50 microscope. The proportion of positively stained cells was calculated as the number of positive cells divided by the total number of cells.

### Protein preparation

The flap tissues were frozen in liquid nitrogen and stored at −80°C until use. The tissues were homogenized in ice-cold isolation solution containing 250 mmol/l sucrose, 10 mmol/l triethanolamine, 1 μg/ml leupeptin and 0.1 mg/ml phenylmethylsulfonyl fluoride. The homogenates were centrifuged at 15,000 × g for 10 min at 4°C to separate the incompletely homogenized tissue. The supernatants were obtained and the protein concentrations were measured using a protein assay kit (Sunbio, Beijing, China). An *N*-glycosidase F Deglycosylation kit (Roche Diagnostics GmbH, Mannheim, Germany) was used to deglycosylate the proteins.

### Western blotting

Total proteins (50 μg/sample) were diluted in 5X loading buffer [0.25 mol/l Tris HCl (pH 6.8), 10% sodium dodecyl sulfate (SDS), 0.5% bromophenol blue, 50% glycerol and 0.5 mol/l dithiothreitol] and boiled for 5 min. SDS-polyacrylamide gel electrophoresis (PAGE) was performed on 12% gradient gels. The proteins were transferred electrophoretically to polyvinylidene difluoride membranes (Millipore, Billerica, MA, USA) pre-treated with methanol and blocked for 1 h at room temperature in Tris-buffered saline with 0.1% Tween-20 containing 5% non-fat dry milk (TBS-T). They were subsequently incubated overnight at 4°C with anti-HMGB1 antibody (1:100) and anti-NF-κB antibody (1:500; both from Santa Cruz Biotechnology, Santa Cruz, CA, USA) in TBS-T containing 5% non-fat dry milk. Following washing in TBS-T, the membranes were incubated with horseradish peroxidase-labeled anti-rabbit antibody (1:3,000; Santa Cruz Biotechnology) for 2–3 h at room temperature. The blots were developed with enhanced chemiluminescence agents (ECL Plus; Sunbio) prior to exposure to X-rays. To confirm equivalent loading of the samples, the same membranes were incubated with anti-β-actin antibody (1:300; Santa Cruz Biotechnology) and visualized by enhanced chemiluminescence as described above. For quantification, films of the western blot analysis were scanned using a Minolta scanner (Konica Minolta, Inc., Tokyo, Japan) and Adobe Photoshop software. The labeling density was quantitated using Lab Works software (UVP, Upland, CA, USA). The values of the relative densities of HMGB1 and NF-κB bands were normalized to the density of actin to represent the quantity of HMGB1 and NF-κB protein, respectively.

### Statistical analysis

Statistical analysis was performed using SPSS version 15.0 (SPSS Inc., Chicago, IL, USA). All quantitative data are expressed as the mean ± standard deviation. A one-way analysis of variance was used to test the significance of differences in HMGB1 and NF-κB western blot analysis, and survival area. The associations between skin injury scores, and the expression of HMGB1 and NF-κB were analyzed by calculating Pearson product-moment correlation coefficients. P<0.05 was considered to indicate a statistically significant difference.

## Results

### HBO-PC increases blood perfusion in epigastric skin flaps

**Figure 1:**
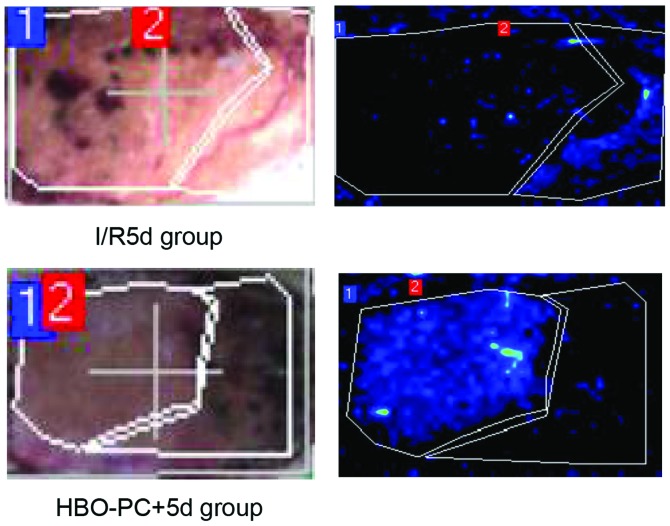
Relative changes in laser Doppler images of skin flap grafts in rats of the I/R5d and HBO-PC+5d groups. (Left) Representative histological images. (Right) Representative laser Doppler images. I/R5d, ischemia followed by reperfusion 5 days following surgery; HBO-PC+5d group, hyperbaric oxygen preconditioning and ischemia followed by reperfusion 5 days following surgery.

LDPI is a noninvasive and relatively new technique for measuring skin blood perfusion (15). LPDI was initially performed 5 days following skin grafting to detect blood perfusion. As shown in Fig. 1, HBO-PC increased blood perfusion (dark blue) compared with the groups with I/R alone (P<0.05). Notably, the LDPI data were consistent with the histological data, which indicated that HBO-PC protects against I/R injury during epigastric skin flap grafting.

### HBO-PC attenuates I/R injury following skin flap grafting

**Figure 2:**
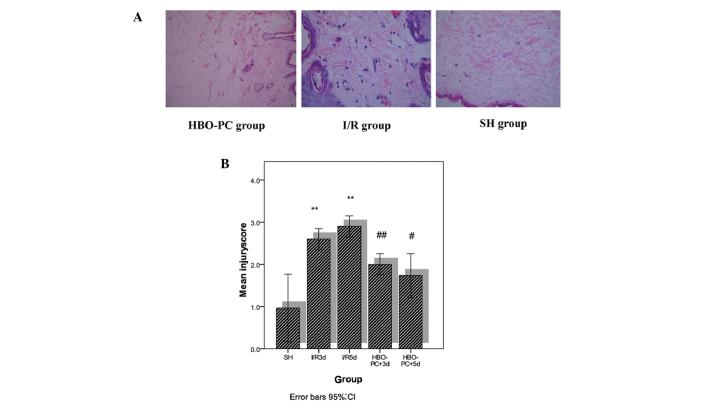
(A) Representative skin flap microscopic images: HBO-PC group rats (left), I/R group rats (middle) and SH group rats (right). (B) Injury scores of the SH group (n=8), I/R3d group (n=8), I/R5d group (n=8), HBO-PC+3d group (n=8), and HBO-PC+5d group (n=8). Data are presented as the mean ± standard deviation ^**^P<0.01 for SH versus I/R groups. ^#^P<0.01 for HBO-PC+5d group versus I/R5d group, ^##^P<0.05 for HBO-PC+3d group versus I/R3d group. SH, sham surgery; I/R3d, ischemia followed by reperfusion 3 days following surgery; I/R5d, ischemia followed by reperfusion 5 days following surgery; HBO-PC+3d, hyperbaric oxygen preconditioning and ischemia followed by reperfusion 3 days following surgery; HBO-PC+5d, hyperbaric oxygen preconditioning and ischemia followed by reperfusion 5 days following surgery.

Following grafting, the skin flaps from the I/R groups exhibited typical edema, effusion and necrosis, and were dark purple in color. However, the skin flaps from the HBO-PC groups exhibited a low level of effusion demonstrated by pink skin flap coloration. Histological examination of the skin flap sections from the I/R groups showed significant edema and congestion (Fig. 2). The injury scores, which were calculated from congestion, epidermis edema and neutrophil infiltration, were significantly higher in the I/R groups compared with the SH group. However, the injury scores of the HBO-PC groups were significantly lower compared with the I/R groups. These data indicate that HBO-PC may attenuate I/R injury during skin flap grafting.

### HBO-PC suppresses HMGB1 and NF-κB expression in skin flaps

**Figure 3:**
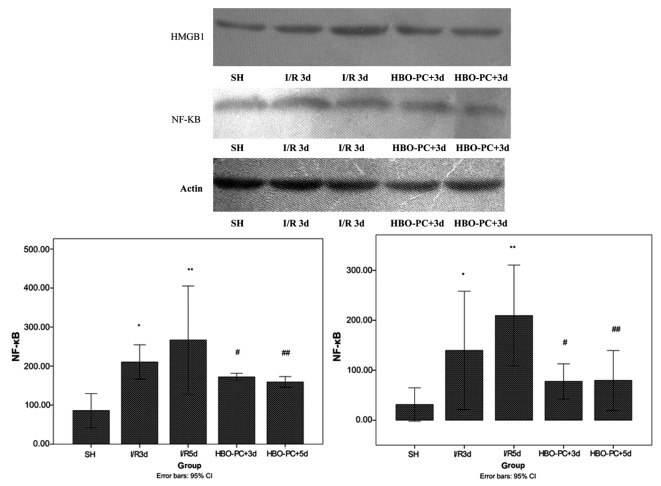
Expression of HMGB1 and NF-κB protein in the SH group (n=7), I/R groups [by I/R3d and I/R5d groups, n=7], and HBO-PC groups (HBO-PC+3d and HBO-PC+5d groups, n=7). Data are presented as the mean ± standard deviation. ^**^P<0.01 for the SH group vs. the I/R5d group, ^*^P<0.05 for the SH group vs. the I/R3d group, ^#^P<0.05 for the I/R3d group vs. the HBO-PC+3d group, ^##^P<0.01 for the I/R5d group vs. the HBO-PC+5d group. HMGB1, high mobility group protein 1; NF-κB, nuclear factor-κ B; .SH, sham surgery; I/R3d, ischemia followed by reperfusion 3 days following surgery; I/R5d, ischemia followed by reperfusion 5 days following surgery; HBO-PC+3d, hyperbaric oxygen preconditioning and ischemia followed by reperfusion 3 days following surgery; HBO-PC+5d, hyperbaric oxygen preconditioning and ischemia followed by reperfusion 5 days following surgery.

**Figure 4:**
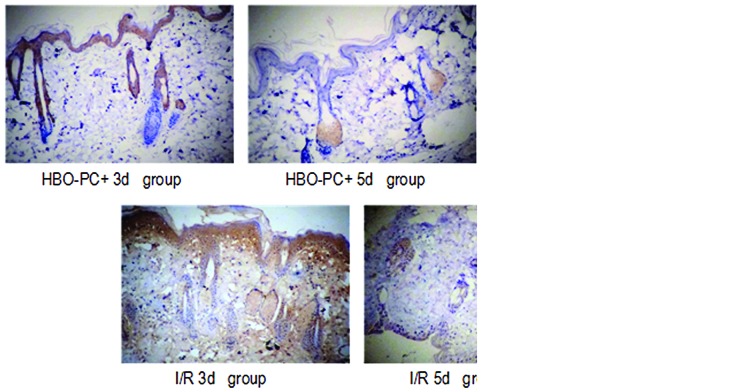
Immunohistochemical localization of NF-κB protein. NF-κB nuclear factor-κ B; SH, sham surgery; I/R3d, ischemia followed by reperfusion 3 days following surgery; I/R5d, ischemia followed by reperfusion 5 days following surgery; HBO-PC+3d, hyperbaric oxygen preconditioning and ischemia followed by reperfusion 3 days following surgery; HBO-PC+5d, hyperbaric oxygen preconditioning and ischemia followed by reperfusion 5 days following surgery.

**Figure 5:**
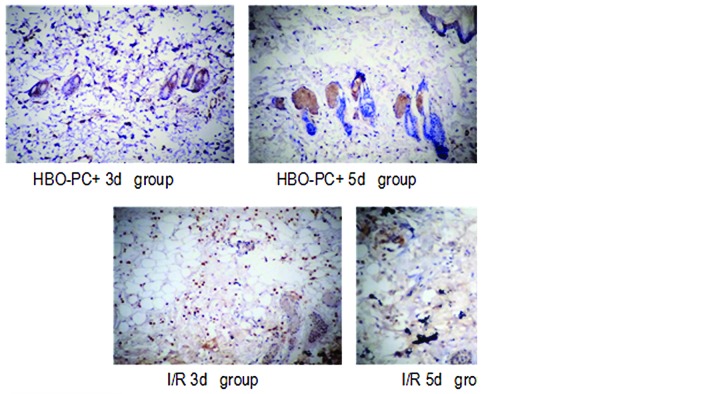
Immunohistochemical localization of HMGB1 protein. HMGB1, high mobility group protein 1; I/R, ichemia/reperfusion.; SH, sham surgery; I/R3d, ischemia followed by reperfusion 3 days following surgery; I/R5d, ischemia followed by reperfusion 5 days following surgery; HBO-PC+3d, hyperbaric oxygen preconditioning and ischemia followed by reperfusion 3 days following surgery; HBO-PC+5d, hyperbaric oxygen preconditioning and ischemia followed by reperfusion 5 days following surgery.

The potential mechanisms underlying the protective effect of HBO-PC were investigated. The expression of HMGB1 and NF-κB were examined by western blotting (Fig. 3) and immunohistochemical staining (Figs. 4 and 5).

HMGB1-positive cells were rarely observed in the skin flap tissue in the SH group. By contrast, there were significantly more HMGB1-positive cells in the ischemic skin flaps in the I/R groups. The percentages of positive staining in the SH group, and the I/R3d and I/R5d were 7.8, 34.5 and 42.3%, respectively. However, HBO-PC significantly attenuated the I/R injury-elicited increase in HMGB1 expression (P<0.01). The percentages of positive staining in the HBO-PC+3d and HBO-PC+5d groups were 31.6 and 28.2%, respectively. There was no significant difference in the numbers of HMGB1-positive cells between the HBO-PC+3d and HBO-PC+5d groups (P>0.05).

The percentages of positive staining for NF-κB in the I/R3d, I/R5d, HBO-PC+3d, and HBO-PC+5d groups were 45.2, 57.9, 37.6 and 31.2%, respectively. Thus, HBO-PC significantly attenuated NF-κB expression (P<0.01).

As shown in Figs. 4 and 5, the expression of HMGB1 and NF-κB were distributed in the cytoplasm and nucleus in the I/R groups and primarily in the cytoplasm following HBO-PC treatment.

Western blotting was performed to further verify the expression of these two proteins. Concordant with the results of immunohistochemical staining, HBO-PC significantly decreased the expression of HMGB1 and NF-κB proteins compared with their expression in I/R injury following skin flap grafting (P<0.01; Fig. 3).

## Discussion

I/R injury is a severe injury that occurs when circulation is re-established following ischemia in skin flap graft surgery. It induces a cascade of pathophysiological changes, including neutrophil influx, interstitial edema and increased permeability, resulting in skin flap necrosis or graft loss (16,17). In the current study, to induce I/R injury, inferior epigastric vessel pedicled skin flaps were designed in which the feeding vessels of the skin flaps were clamped and removed 3 h later. I/R-induced reactive oxygen species (ROS) initiate subsequent injury (18,19). The formation of ROS contributes to the activation of adhesion molecules, which leads to leukocyte infiltration (20) and causes a series of changes that impair microcirculation (21,22).

Accumulating evidence has demonstrated that HBO-PC is a promising strategy for protecting cells against I/R injury (23–25). HBO-PC increases the levels of antioxidants, including GSH and superoxide dismutase (SOD) to prevent ROS-induced lipid peroxidation in membranes (26). Li *et al* (27) further confirmed that HBO-PC improves ROS scavenging. It was hypothesized that an endogenous antioxidative protective pathway is initiated through HBO-PC when patients are exposed to ROS during cardiopulmonary bypass surgery. However, the molecular mechanism of HBO-PC remains unknown. ROS are an initiating factor, while the ROS scavenging ability of HBO-PC may inhibit the interactions between specific I/R-associated mediators and their corresponding receptors. Thus, in the current study, it was hypothesized that HBO-PC attenuates I/R injury following skin flap grafting by mediating two important inflammatory mediators, HMGB1 and NF-κB, which inhibits the subsequent inflammatory reaction.

HMGB1 is a highly conserved non-histone DNA-binding protein that stabilizes DNA and is crucial in the inflammatory response during I/R injury (28). HMGB1 may be passively released from necrotic and damaged cells or actively secreted from macrophages and monocytes (29–32). Previous reports demonstrated that HBO treatment is appropriate for decreasing HMGB1 levels in patients with cerebral injury (33). In the present study, the elevated expression of HMGB1 protein in the skin flap tissue of the I/R groups was attenuated by HBO-PC. The majority of samples from the I/R groups showed marked HMGB1 cytoplasmic staining. Thus, it was hypothesized that HBO-PC protects membrane proteins from lipid peroxidation through ROS scavenging. The I/R-elicited ROS may promote HMGB1 translocation from the nucleus to the cytoplasm as well as its release into the extracellular space. However, this pathway is blocked by HBO-PC through the removal of ROS, which terminated I/R-induced cell necrosis and macrophage activation.

As a transcription factor, NF-κB controls the expression of pro-inflammatory proteins. Under normal conditions, NF-κB is in an inactive state and mainly located in the cytoplasm. Activated NF-κB translocates from the cytoplasm to the nucleus through nuclear pores. NF-κB subsequently combines with the K structure area of target genes, initiating transcription of the corresponding genes encoding inflammatory mediators, including tumor necrosis factor and interleukin-6 (34–36). Concordant with the results reagrding HMGB1, the expression of NF-κB in the I/R group was higher compared with that in the SH group. These changes in NF-κB expression paralleled those of HMGB1 expression, suggesting a possible association between HMGB1 and NF-κB activation in the I/R process following skin flap grafting. Wang *et al* (37) reported that tanshinone IIA effectively decreases tissue injury under cerebral ischemic conditions through the downregulation of the NF-κB activation pathway. The current data suggest that HBO-PC inhibits the release of HMGB1 into the extracellular space, and is likely to prevent HMGB1 from binding to cell-surface receptors, including toll-like receptor 4 (TLR4) and blocking MyD88-dependent transduction pathways. The inactivation of the TLR4 pathway inhibits the translocation and DNA binding activity of NF-κB, consequently decreasing pro-inflammatory cytokine production. Immunochemical analysis revealed that NF-κB expression in the HBO-PC group was significantly reduced in the cytoplasm and nucleus. In agreement with this hypothesis, a previous study demonstrated that the expression of TLR4 and NF-κB protein is increased by I/R injury and downregulated by ischemic preconditioning through the mediation of the TLR4/NF-κB pathway (38).

In the present study, LDPI was used to monitor skin blood perfusion in different experimental groups. Skin blood flow to surface tissue was significantly higher in the HBO-PC groups compared with the I/R groups. Therefore, this technique provides invaluable evidence demonstrating that HBO-PC can improve microcirculation of skin flap grafts.

On the basis of the results of the current study, it was concluded that HBO-PC effectively promotes the survival of skin flap grafts by eliciting an endogenous protective mechanism. HBO-PC reduces HMGB1 and NF-κB expression in skin flaps, suggesting that the inhibition of HMGB1 and NF-κB expression is associated with blocking the TLR4/NF-κB pathway during I/R injury. In addition, HBO-PC inhibits the inflammatory response at the early stage of I/R injury, providing an attractive therapeutic avenue for skin graft surgery.
